# A real-time end-to-end detector for detecting surface defects on oversized rings

**DOI:** 10.1371/journal.pone.0330031

**Published:** 2025-08-12

**Authors:** Junwei Liu, Lihua Zhang, Shuguang Chen, Yesong Wang, Binbin Wu, Junjun Dong

**Affiliations:** 1 Jiangsu University of Science and Technology, Zhengjiang, China; 2 China Academy of Machinery Ningbo Academy of Intelligent Machine Tool Co. Ltd, Ningbo, China; Industrial University of Ho Chi Minh City, VIETNAM

## Abstract

Oversized rings in wind turbines are regarded as crucial components because they often serve as the main load-bearing and connector structures. Surface defects on these rings can disrupt the normal operation of the entire unit. Detecting surface defects on oversized rings in wind turbine generators (WTGs) is highly challenging due to the huge ring size and small target defects, which will cause the detection process to be very time-consuming and difficult to achieve the expected accuracy. To address this challenge, we propose a new lightweight multiscale high-efficiency detector (LMHD) that balances accuracy and model size. The framework utilizes RepViT as the detection backbone and incorporates a bi-directional feature pyramid network (BiFPN) in the neck to achieve bi-directional feature transfer and aggregation. Additionally, it includes a new lightweight, efficient, multi-scale cross-stage partition module called the Diverse View Group Shuffle Cross Stage Partial Network (DVOV-GSCSPM), which employs a rational architecture and multiscale information fusion to ensure that the overall model is lightweight while maintaining a rich gradient flow. Self-Calibrated Convolutions (SCConv) and Efficient Local Attention (ELA) modules are introduced into the neck network to reduce computational complexity and the number of parameters while ensuring model accuracy. Ultimately, we incorporate the Powerful-IoUv2 loss function to enhance the rate of model convergence and generalization capabilities. The model is experimentally validated on the public dataset NEU-DET, achieving a detection accuracy of 87.0% with 70.4 frames per second (FPS).

## 1. Introduction

Currently, the world is vigorously developing clean energy, and wind energy represents the most mature technology in this field, with significant large-scale development potential and commercial prospects. Oversized rings are crucial components of wind turbines for carrying and connecting. They must meet long-term service requirements, typically exceeding 20 years [1]. During the molding process, near-surface defects like porosity, cracks, inclusions, and folds are easily produced. These defects directly impact the service performance and lifespan of the components. To ensure product quality, it is essential to conduct defect detection on these ring parts. Presently, manual inspection remains the primary method for detecting surface defects on ring parts. However, manual inspection results are often influenced by inspectors subjectivity, leading to visual fatigue and potential errors. Manual inspection is prone to oversight, misidentification, and lacks consistency in results.

Moreover, due to the large size of these ring parts, manual inspection efficiency is relatively low. Therefore, there is a need to automate the detection of surface defects on oversized rings using computer vision technology.

Traditional methods for detecting steel surface defects suffer from human subjective judgment, high cost, low efficiency, error-prone results and safety risks. Recent advancements in product-specific defect detection methods, such as eddy current nondestructive inspection [2], laser inspection [3], and liquid penetration inspection [4]. However, these methods face challenges such as usage limitations, difficulty in defect classification, susceptibility to light and complex background interference, which hinder accurate and efficient detection. In contrast, deep learning methods can autonomously learn defect samples and extract defect features from images to effectively detect various types of defects. This greatly improves detection efficiency and reduces the waste of human and cost resources in the defect detection process. Deep learning methods have great potential for detecting surface defects in steel [5]. Mainstream surface defect detection methods based on deep learning include fully supervised models, unsupervised models, and other models such as representation-based learning, metric-based learning, normal sample- based learning, semi-supervised models, and weakly supervised models.

Despite the potential benefits of deep learning in defect detection, its application in industrial settings is limited by challenges and requirements [6]. The detection of steel surface defects is a challenging task due to the complexity and variability of steel surface defects, as well as the disturbances in industrial environments [7]. Not only are computational resources limited in industrial environments, but defect detection algorithms also need to have high accuracy, efficiency, and real-time performance [8]. Accurately detecting defects of the same type with large variations remains a serious challenge, as the detection of defects of different sizes varies greatly in terms of accuracy and recall. The current mainstream method addresses this issue by extracting multi-scale feature information, but balancing the retention of semantic and spatial feature information requires further research. Increasing the number of convolutional layers may lead to the loss of important spatial features, resulting in inaccurate detection of small defects. Moreover, a higher number of model parameters can improve detection accuracy, but it may pose challenges in deployment. Therefore, the algorithm needs further optimization to retain more spatial feature information while keeping the model lightweight. Deep learning still has room for improvement in detection accuracy for accurate real-time identification of small targets with surface defects on oversized rings. Challenges include difficulties in detecting complex and subtle defects, significant loss of feature information, and instances of misdetection and omission.

To address these issues, the proposed LMHD algorithm for recognizing small targets in oversized ring images makes the following significant contributions:

1. To ensure that the model is lightweight while achieving excellent detection performance, this paper employs the RepViT network as the backbone to extract semantic and spatial information from images.
2. This paper proposes a DVOV-GSCSPM module to extract multi-scale information, incorporating global and local feature information to better capture the contextual information of nodes.
3. The neck network utilizes a weighted feature pyramid network (BiFPN), which employs a learnable feature fusion operation that enables different levels of feature maps to be adjusted to each other, thereby significantly enhancing target detection performance.
4. By integrating the SCConv and ELA attention mechanisms in the neck network, the combination of convolution and attention mechanisms enables the model to capture long- distance dependencies and correlations, better integrate global information, and process features more comprehensively and finely, thus improving the overall performance and expressive ability of the model.
5. The Powerful-IoUv2 loss is incorporated into this model to address the issue of anchor box expansion during the regression procedure slowing down model convergence, aiming to enhance the model’s robustness and generalization.
6. In order to verify the capability of the algorithm in this paper, several experiments were conducted on the NEU-DET dataset, and the results proved that the proposed algorithm achieves the SOTA detection accuracy in the detection model.

## 2. Related works

### 2.1 Deep learning based target detection

In order to replace manual inspection, the use of computer vision techniques to automatically detect surface defects in oversized rings is desired. Recently, deep learning has demonstrated significant potential in the fields of classification and detection, offering an effective solution for identifying surface defects in oversized rings. The development of deep learning techniques began with the pioneering convolutional neural network architecture LeNet-5 [9], followed by the groundbreaking AlexNet [10], and subsequently advanced through algorithms such as ResNet [11], VGG, MobileNet, and EfficientNet [12]. Zhou et al. [13] enhanced the kernel and its size in the standard LeNet-5 model and integrated it with the particle swarm optimization algorithm to propose an improved LeNet-5 model for fault detection in liquid piston pumps. Marques [14] developed a diagnostic system utilizing EfficientNet, significantly improving the accuracy of medical decision-making systems. Michele et al. [15] fine- tuned the pre-trained MobileNet neural network and investigated the applicability of the MobileNet V2 deep convolutional network. The results indicated that the classification accuracy of MobileNet V2 reached 100%. As network architectures have become more complex, their feature extraction capabilities have strengthened. Deep learning networks have demonstrated exceptional performance in image recognition, particularly in object detection, where deep learning-based feature extraction networks have achieved significant progress. Deep learning-based image recognition algorithms can be broadly categorized into two types: one-stage and two-stage algorithms. Based on their algorithmic processes, object detection algorithms can be further divided into two categories: two-stage algorithms, represented by Faster R-CNN [16], which detect objects through generating candidate regions and refining bounding boxes; and one- stage algorithms, represented by SSD [17] and YOLO [18], which classify and refine bounding boxes directly from anchor points. The two approaches have distinct strengths: the two-stage algorithm typically achieves higher detection accuracy at the cost of slower processing speed, whereas the single-stage algorithm achieves faster processing speed at the expense of accuracy, often surpassing the two-stage algorithm in speed.

### 2.2 Two-stage detection framework

Two-stage target detection algorithms generally comprise two primary stages. The first stage involves generating candidate target regions, typically using the Region Proposal Network (RPN) or similar methods. The second stage involves classifying and refining the localization of these candidate regions. Two-stage detection algorithms are designed to achieve high recognition accuracy. For instance, Ren et al. [19] proposed a structural visual detection method based on the Faster Region-based Convolutional Neural Network (Faster R-CNN) to enhance the network’s ability to distinguish between various types of defects. Shi et al. [20] utilized the ConvNeXt architecture for feature extraction in Faster R-CNN, enhancing the network’s ability to suppress complex background features and improve strip detection accuracy. Dual-stage algorithms typically excel in accuracy for target detection tasks, delivering high-quality results. However, due to the requirement for two independent stages, these dual-stage algorithms often necessitate more computational resources and time, resulting in relatively slower inference speeds. Consequently, two-stage detection frameworks are not suitable for industrial production settings.

### 2.3 One-stage detection framework

To meet industrial real-time detection requirements, many researchers have investigated single-stage detection algorithms. Lu et al. [21] introduced an enhanced WSS-YOLO detection algorithm, utilizing the WIoU loss to mitigate the challenges posed by low-quality samples. They also developed the C2f-DSC module, integrating Dynamic Serpent Convolution (DSC) to augment the model’s feature extraction capabilities and enhance steel detection. Gao et al. [22] introduced the SRN-YOLO detection model, enhancing YOLOv7 with the Split Residual Convolutional Network (SResNet) to capture gradient feature information. They also developed the Re-fused Feature Pyramid Network (RFPN) to preserve additional positional information about the target. Wang et al. [23] enhanced the multi-scale YOLO-v5 algorithm and developed the Multi-Scale module to improve steel detection performance. Generally, current methods for detecting defects on steel surfaces primarily concentrate on medium and large-scale defects, which manifest in various forms, posing challenges in accurately localizing and classifying the defective regions. However, the detection performance is suboptimal for some defects in small and unclear states. Regarding industrial deployment, larger model sizes are more conducive to improving detection accuracy, albeit with deployment limitations, whereas lightweight detection models are more suitable for practical industrial applications. Based on the above insights, we propose a new Lightweight Multiscale High-Efficiency Detector (LMHD) designed to meet industrial requirements for detecting surface defects on oversized rings.

## 3. Methods

### 3.1 LMHD detection model

**Fig 1:**
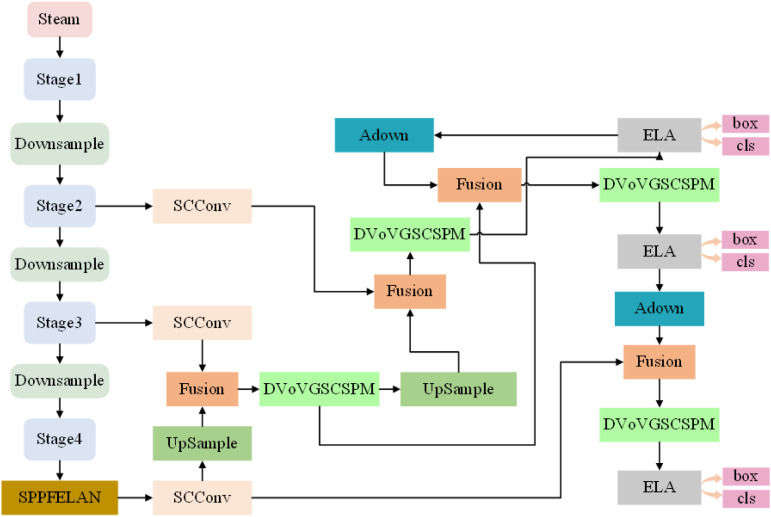
Schematic diagram of the LMHD modeling framework.

To improve the accuracy of surface defect detection in oversized rings of wind turbines, we introduce a new lightweight target detection network called LMHD. The overall framework of the LMHD detection model is depicted in Fig 1, consisting of four phases: feature extraction, feature fusion, detection frame inference, and post-processing. The defective images of the oversized rings are fed into the LMHD model and downsampled through the backbone network. The resulting four output feature maps are then passed from the backbone network to the neck network. The neck network utilizes a weighted bidirectional feature pyramid network (BiFPN) to extract feature information related to the surface defects of the oversized rings, thereby enhancing the network’s multi-scale feature extraction capability and field of view. The up-sampled feature maps are concatenated with the coarser- grained feature maps in the backbone network to enhance the model’s multi-scale feature extraction capability. Additionally, this study introduces a novel lightweight and efficient multi-scale cross-level component module (DVOV-GSCSPM), inspired by a single aggregation module. Upon receiving the output feature maps at three different scales from the neck network, the detection head network generates the coordinates of all candidate bounding boxes using various anchor box sizes. Following these steps, the regions potentially containing defects on the surface of the input oversized rings are identified, aiding in the detection of surface defects on the oversized rings.

### 3.2 RepViT backbone network

**Fig 2:**
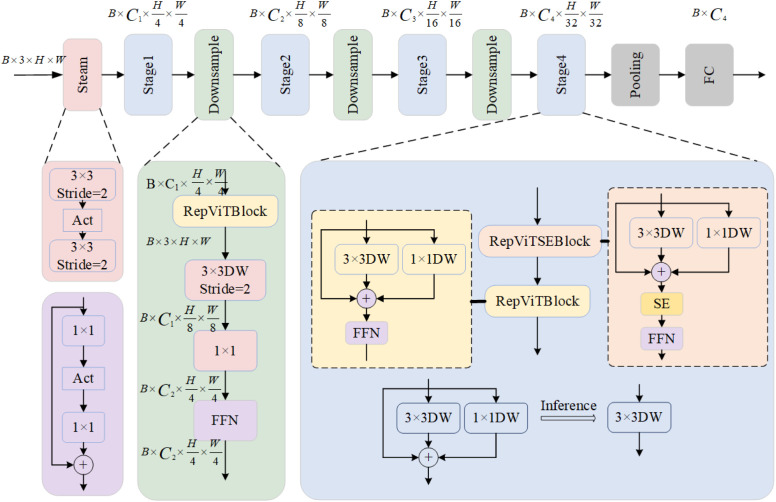
Repvit network structure.

Making large models practical and ensuring high accuracy and low latency, it is crucial to lightweight them. Lightweight networks are characterized by fewer parameters, computations, and shorter inference times compared to heavyweight networks. They are more suitable for scenarios with limited storage space and power consumption, such as edge computing devices like mobile embedded devices. The lightweight target detection network model reduces the deployment cost in resource-constrained environments, such as different devices, while also accelerating inference speed and enhancing real-time performance and user experience. Various efficient convolutional neural network attention mechanisms and methods, including designing convolutional kernels of varying sizes, inter-module short- circuiting, and stacked convolution, are employed in the network to enhance visual task performance. Increasing the depth of the network and stacking various modules significantly enhances the network’s ability to represent features. However, this improvement also brings negative consequences, such as a substantial increase in computational complexity, resulting in longer training times and higher inference latencies, posing challenges in meeting the real- time requirements of some applications. To enhance the lightweight nature of the defect detection model for oversized rings of wind turbines, this paper proposes using the latest lightweight backbone, RepVit [24],as the underlying framework, illustrated in Fig 2. The RepVit network can be tuned to a lightweight CNN through layer-by-layer micro-design, which includes selecting the suitable convolutional kernel size and optimizing the position of the squeeze-and-excitation (S&E) layer, resulting in substantial improvement in model performance. The shallow network employs a convolutional extractor to extract features to a deeper downsampling layer. Initially, it utilizes a 1 × 1 convolution to adjust the channel dimensions, followed by connecting the inputs and outputs of the two 1 × 1 convolutions with residuals to create a feed-forward mesh termination. Additionally, a RepVit block is added at the front to further deepen the downsampling layer, enhancing model accuracy more efficiently.

RepViT is structured into three stages. The backbone network consists of 3 × 3 convolutional stacks with a stride of 2. Compared to MobileNetV3 [25], latency is reduced despite an increase in the number of convolutional kernels. Fine-tuning RepViT networks to lightweight CNNs through layer-by-layer microdesign, which includes choosing the right convolutional kernel size and optimizing the placement of the Squeeze-and-Excitation (SE) layer, can significantly enhance model performance. The shallow network employs a convolutional extractor to move to a deeper downsampling layer, initially using a 1 × 1 convolution to adjust channel dimensions. Additionally, a RepViT block is added at the start to further deepen the downsampling layer, aiming to enhance model accuracy more effectively and mitigate image information loss due to resolution degradation during feature extraction. In RepViT, the deep downsampling layer includes the RepViT block, a deep separable convolution, and an FFN. RepViT offers six variants: RepViT-M0.6/M0.9/M1.0/M1.1/M1.5/M2.3, each configured based on channel numbers at each backbone network stage and block counts per stage. This paper advocates using RepViT-M0.6 as the model.

In summary, RepViT is an innovative lightweight network architecture tailored for edge devices. It achieves high accuracy while retaining a lightweight network structure.

Additionally, efforts have been made to further reduce the number of parameters in the LMHD model.

### 3.3 Variety of view group shuffle cross stage partial network

Current state-of-the-art (SOTA) detectors employ a multi-scale detection approach, enabling comprehensive object detection across different sizes. While the original network performs well in most cases, its lack of sensitivity to small-sized defects on oversized rings in wind turbines often leads to false detections. Typically, previous methods tackle this issue by introducing new detection scales, which, however, increases model parameters and computational demands.

Building upon the above analysis and inspired by the single aggregation module (VoVGSCSP) [26], this study introduces a new lightweight multi-scale feature aggregation module (DVoVGSCSPM). This module aims to achieve higher parameter utilization and computational efficiency, designed for flexible integration of various computational modules to enhance detection of small-target defects on oversized rings.

**Fig 3:**
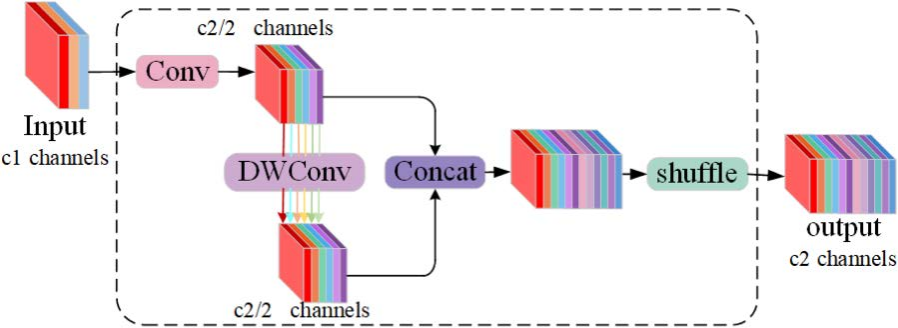
GSConv module structure.

**Fig 4:**
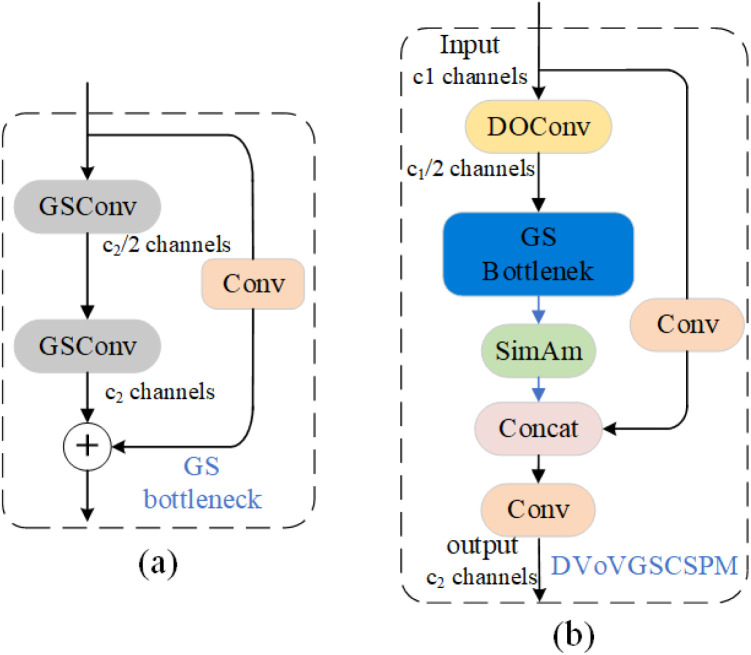
DVoVGSCSPM network structure. **(a)** Structure of GS bottleneck module; **(b)** Structure of DVoVGSCSPM module.

To reduce model parameters and computational complexity, a lightweight convolution module, GSConv (Group-shuffle Convolution), has been integrated into the structure, as shown in Fig 3. GSConv conducts depth-separable convolution on data from standard convolution and then reorganizes channel information to produce results. By maintaining hidden connections between channels, GSConv efficiently transfers relevant information among features, thereby preserving semantic information more effectively. This enables GSConv to uphold high prediction accuracy and semantic expressiveness while accelerating predictions. GSConv implements feature information reuse, enabling the model to achieve lower computational complexity and higher detection accuracy, significantly enhancing the balance between accuracy and speed. As shown in Fig 4(a), a GS bottleneck design is introduced leveraging the GSConv module. The VoVGSCSP module is derived from the GSConv module’s design aggregation. Consequently, DOConv convolution and parameter-free attention mechanism [27] (SimAM) are integrated. Combining convolutionally extracted multi-scale features with the attention mechanism enables the model to better capture target information across scales, enhancing its adaptability to variations in scale. The DVoVGSCSPM module simplifies computation and network structure while maintaining adequate accuracy, as shown in Fig 4(b).

**Fig 5:**
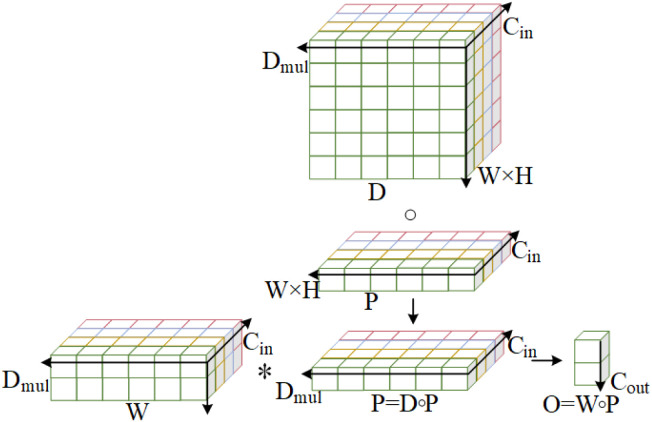
Structure of depthwise over-parameterized convolution module.

In the CBS module, the input feature map is divided into two branches by a convolutional layer. The first branch reduces channels using a pointwise convolutional layer and then generates a binary mask via a sigmoid function. The second branch creates a feature map through a residual block. Finally, a compressed feature map is produced by element-wise multiplication of the binary mask and the feature map. However, this compressed feature map lacks sufficient information from the original feature map and disregards interactions between feature spaces. This poses a challenge in extracting critical defect features from surface images of oversized rings, ultimately reducing the model’s feature extraction capability and detection accuracy. To address this issue, we propose using a depth over- parameterized convolutional layer (DOConv) [28] instead of the CBS module, as shown in Fig 5. DOConv consists of two parts: depth convolution and traditional convolution. Unlike traditional convolution, each convolution kernel in depth convolution operates on a single channel. Depth convolution can be used as a spatial convolution on each channel separately. Therefore, it outputs the same number of feature maps as input channels without dimensionality expansion.

### 3.4 DVoVGSCSPM module

In contrast to the original CBS module, DOConv employs additional depthwise convolution (DWConv) for layer enhancement, allowing each input channel to be convolved with a distinct 2D kernel. Introducing learnable parameters, the combination of two convolutions results in over-parameterization, allowing linear operations to be represented by a single convolutional layer. Traditional convolution involves nonlinear layers, and increasing the number of nonlinearities enhances the neural network’s representation without causing overfitting.

Additionally, DOConv accelerates the network training process by combining two convolutional layers. It enriches the feature map’s semantic information and enhances the network’s feature extraction capability without increasing computational requirements during inference, significantly improving CNN performance. This improvement boosts training speed and accuracy without requiring additional computation during inference. The inference formula is presented in Equation (1).


$$O_{C_{out}}=\sum_{i}^{(M\times N)\times C_{in}}W_{C_{out^{i}}}P_{i}$$ (1)

M and N represent the number of pixels in the height and width of the input image, $C_{in}$ denotes the number of channels in the input tensor, and $C_{out}$ refers to the number of channels in the DOConv output tensor.

**Fig 6:**
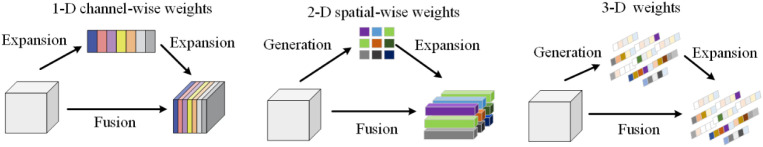
Attention mechanism without reference.

Additionally, to develop a module for extracting global feature information and interactions, we propose a parameter-free attention mechanism (SimAM). SimAM is a parameter-free attention mechanism that identifies and enhances important feature regions by calculating the similarity between different locations in the feature map. It can effectively improve the module’s ability to extract global feature information without increasing computational cost, and enhance the expressive power of the network model, thereby improving detection accuracy.The overall structure of SimAM is illustrated in Fig 6. The input feature map has C channels and a size of H*W. The energy function generates 3D weights, which are normalized using the Sigmoid function. The desired neurons’ weights are multiplied by the original feature map’s features to obtain the final output feature map. Unlike traditional channel and spatial attention modules, the SimAM module does not introduce any parameters to the original network. Incorporating SimAM attention significantly addresses overfitting and enhances the network’s nonlinear representation. Instead of performing internal convolution operations, SimAM calculates 3D attention weights of the feature maps based on channel weights computed from the energy function. To achieve improved behavioral attention, each neuron’s significance is assessed by minimizing the energy function and deriving a fast closure solution. This minimizes the need for extensive reorganization since most operators are selected based on the energy function’s solution.

Information-rich neurons typically exhibit distinct firing patterns compared to peripheral neurons. Additionally, firing neurons frequently inhibit peripheral neurons, a phenomenon known as spatial location inhibition. Consequently, neurons exhibiting spatial location inhibition have garnered more interest. The simplest method to identify these neurons is by assessing the linear separability between a target neuron and other neurons. An associated energy function has been proposed and utilized to determine the significance of each neuron, as depicted in Equation (2).


$$e_{t}\left(\omega_{t},b_{t},y,x_{i}\right)={\left(y_{t}-\hat{t}\right)}^{2}+\frac{1}{M-1}\sum_{i=1}^{M-1}{\left(y_{0}-\hat{x_{i}}\right)}^{2}$$ (2)

Where $\hat{t}=w_{t}t+b_{t}$, $x_{i}=w_{t}x_{i}+b_{t}$, t and $x_{i}$denote the desired neurons and other neurons within the channel of the input feature $X\in R^{C\times H\times W}$. i denotes the spatial dimensionality index, and M denotes the number of neurons on the channel (𝐻 × 𝑊 denotes the size).

Meanwhile, $\omega_{t}$ and $b_{t}$ refer to the weight and bias transformations, respectively. Separately, in order to obtain linear separability between the target neuron t and all other neurons in the same channel, we minimize the equation as in Equation (2). Ideally, when $\hat{t}=y_{t}$、$\hat{x_{i}}=y_{0}$ and $y_{t}\neq y_{0}$, the value of the energy function is minimized to 0, and the linear differentiability is the strongest. Equations (3)–(5) are the specific reasoning process for minimization. A binary classification of labels 1 and −1 is used for 𝑦𝑡 and 𝑦0, combined with the regularization term in Eq. (2). The final energy function can be expressed as follows.


$$e_{t}\left(\omega_{t},b_{t},y,x_{i}\right)=\frac{1}{M-1}\sum_{i=1}^{M-1}({-1-(w_{t}t+b_{t}))}^{2}+{\left(1-\left(w_{t}t+b_{t}\right)\right)}^{2}+\lambda \omega_{t}^{2}$$ (3)

Theoretically, each channel has a set of M energy functions and the analytical solution of the above equation is as follows.


$$\begin{array}{c} w_{t}=-\frac{2\left(t-\mu_{t}\right)}{{\left(t-\mu_{t}\right)}^{2}+2\sigma_{t}^{2}+2\lambda } \end{array}$$ (4)

$$\begin{array}{c} b_{t}=-\frac{1}{2}\left(t+\mu_{t}\right)\omega_{t} \end{array}$$ (5)

where $\mu_{t}=\frac{1}{M-1}\sum_{i=1}^{M-1}x_{i}$, $\sigma_{t}^{2}=\frac{1}{M-1}\sum_{i}^{M-1}{\left(x_{i}-\mu_{t}\right)}^{2}$ are the mean and variance computed for all the neurons in the channel except t. The solutios of Eqs. (4) and (5) are obtained on a single channel, so it is reasonable to assume that all pixels on a single channel follow the same distribution. the minimum energy function $e_{i}^{*}$ for the ith neuron in the SimAM module isshown below.


$$\begin{array}{c} e_{i}^{*}=\frac{4\left({\hat{\sigma }}^{2}+\lambda \right)}{{\left(t_{i}-\hat{\mu }\right)}^{2}+2{\hat{\sigma }}^{2}+2\lambda } \end{array}$$ (6)

where λ is a hyperparameter that adjusts the size of similarity and affects the distribution of attentional weights. $t_{i}=\sum_{i=1}^{n}\omega_{i}x_{i}+b$ is the i neuron input to the feature map on a single channel, $\hat{\mu }=\frac{1}{M}\sum_{i=1}^{M}x_{i}$ and ${\hat{\sigma }}^{2}=\frac{1}{M}\sum_{i=1}^{M}{\left(x^{i}+\hat{\mu }\right)}^{2}$ represent the mean and variance of all neurons in a single channel. The lower the energy $e_{i}^{*}$ the more distinct the difference between neuron i and its surrounding neurons, the more important it is. Equation (6) aims to quantify the similarity between different locations on the feature map by calculating the energy function to identify feature maps that are significantly different in the channel and spatial domains. And refine the output feature map $\tilde{X}$ by scaling operator as shown in Equation (7):


$$\begin{array}{c} \tilde{X}=sigmoid\left(\frac{1}{E}\right)⊙X \end{array}$$ (7)

where E groups all $e_{i}^{*}\;$in the channel and spatial dimensions.

The efficacy of neuronal information output is enhanced by utilizing closed solutions of the energy function in behavioral feature detection. The newly introduced SimAM module exemplifies flexibility and effectiveness, enhancing the network model’s expressiveness and enabling the network to prioritize regions of interest in behavioral detection. Consequently, this improves the model’s detection accuracy.

After the SimAM module, branch 2 proceeds with a conventional convolution operation, followed by concatenation with the feature map. This is achieved by incorporating concatenation and additional feature fusion strategies, which enhance the detection of small targets while reducing computational expenses. Implementing the DVoVGSCSPM module in the LMHD neck reduces parameters and computations while enhancing detection accuracy.

### 3.5 Multi-scale neck network design

**Fig 7:**
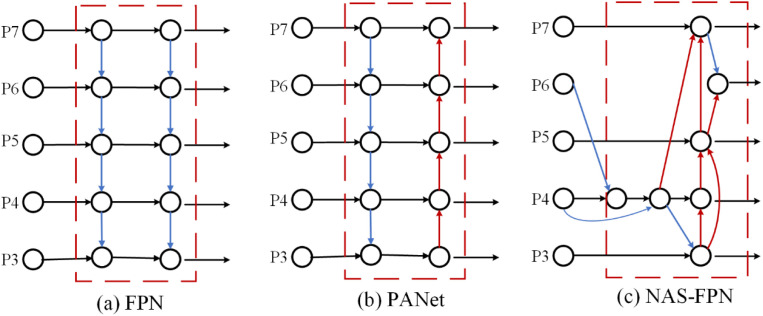
Diagram of different FPN structures. **(a)** FPN introduces a top-down pathway to fuse muli-scale features from level 3 to7(P3 – P7); **(b)** PANet adds an additional bottom- up pathway on top of FPN; (C) NAS-FPN use neural architecturesearch to find an irregular feature network topology and then repeatedly apply the same block.

Early detectors used backbone networks solely to extract pyramid feature layers, with category and location predictions executed in the final layer. To enhance detection accuracy, fusing multi-layer extracted backbone network features is crucial. Outputs from various convolutional layers have different meanings: higher levels offer powerful semantic information but with less detail due to lower resolution, while lower levels have higher resolution but contain excessive noise due to numerous convolutions. Kaiming He et al. first introduced the Feature Pyramid Network (FPN) [29], a feature fusion network to address multi-scale information fusion in object detection. However, FPN supports only top-down feature transfer, making it difficult to convey low-level information to the final layer. To further enhance multi-scale feature fusion, Liu et al. proposed the Path Aggregation Network (PANet) [30], introducing bottom-up paths to FPN to enable more comprehensive information transfer and improve detection performance. PANet provides bidirectional fusion in the feature pyramid, merging shallow and deep semantic information through direct addition. The Google Brain team’s weighted BiFPN [31] (Bidirectional Feature Pyramid Network) creates top-down and bottom-up bidirectional channels based on efficient bidirectional cross-scale connectivity and weighted feature fusion. Information from different backbone scales is unified through up-sampling and down-sampling. Horizontal connections minimize feature loss caused by too many network levels. Various FPN structures are shown in Fig 7.

To enable more effective integration of feature information at different scales, this study employs the BiFPN network as the fusion layer to extract and combine specialized data. Liu et al. [32] proposed a different solution from my method with a different application area, but realized similar functionality and improved the extraction capability of the network. The designed multi-scale necking network greatly improves the model’s ability to extract feature information of defects at different scales in an image, enabling the model to outperform state-of-the-art models in image processing tasks. The extracted features P2, P3, and P4 from the backbone network at three different scales are fed into the BiFPN structure for multiscale feature fusion. The BiFPN assigns each input feature map using fast normalized fusion with learnable weights, allowing the feature fusion nodes in the head network to learn the importance of each input feature through backpropagation during training. Refer to Equation (8) for details on the computation of fast normalized fusion.


$$o=\sum_{i}\frac{\omega_{i}}{\epsilon +\sum_{j}\omega_{j}}\cdot I_{i}$$ (8)

Where o is the weighted feature map, $I_{i}$ is the feature map of the input fusion node, $\omega_{i}\;$and $\omega_{j}$ are the learnable weights, and ϵ=0.001 is to avoid excessive fluctuation of values.

**Fig 8:**
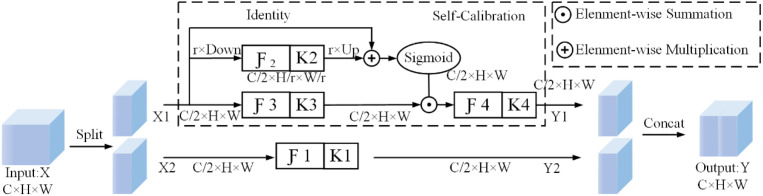
Adaptive convolution structure diagram.


In the interest of facilitating the effective collection of rich contextual information pertaining to each spatial location, it is necessary to adopt a methodology that allows for the comprehensive integration of the neck network,this paper adopts Self-Calibrated Convolutions (SCConv) [33] for the acquisition of feature information from the backbone network to the neck network, as shown in Fig 8. For conventional convolution, suppose the input features are X and the output features are Y, then the conventional 2D convolution consists of a set of filter collections. At this point the description of the conventional convolution is shown into Equation 9.

$$y_{i}=k_{i}*X=\sum_{j=1}^{C}k_{i}^{j}*x_{j}$$ (9)

The input is C×H×W, and the dimension of the convolution kernel k in a convolution where the output channel is also C×H×W. The convolution kernel learning patterns for such convolutions share similarities. Additionally, the field of view at each spatial location in the convolutional feature transform is primarily controlled by a predefined kernel size, and networks comprising stacks of such convolutional layers lack large receptive fields to capture sufficient high-level semantic information. Both drawbacks may result in a less discriminative feature map. Moreover, SCConv convolution can adaptively construct remote spatial and inter-channel dependencies around each spatial location using self-correcting operations, treating the surrounding information environment as an embedding from a low-resolution latent space as an input to the response from the original scale space, and modeling inter- channel dependencies. Therefore, it is possible to practically expand the receptive field of a convolutional layer with self-calibration, which compensates for the shortcomings of a conventional convolutional receptive field. The self-calibration operation does not collect the global context, but only considers the context around each spatial location, avoiding irrelevant information. Additionally, the self-calibration operation can encode multi-scale information, fully extracting the multi-scale features of the surface defects on the oversized rings SCConv convolution increases the receptive field of a CNN through feature map downsampling. Each spatial location can be fused with information from two different spatial scale spaces through a self-calibration operation, augmenting the convolutional receptive field via intrinsic feature communication. This establishes remote spatial and inter-channel dependency relationships, enhancing the diversity of the output features. Without introducing any parameters and complex overhead, the feature information of surface defects on oversized rings is greatly preserved, improving the model’s detection performance.

Specifically, SCConv first inputs a feature map X of size C*H*W and splits it into two $X_{1}$, $X_{2}$ of size C/2*H*W; In the second step, the convolutional kernel K has a dimension of C*C*H*W, and K is divided into four parts, each with a different role, denoted as $K_{1}$,$K_{2}$,$K_{3}$,$K_{4}$, which all have a dimension of C/2*C*H*W, to collect different types of contextual information. ($K_{1}$,$K_{2}$,$K_{3}$,) A self-correcting operation is performed on $K_{1}$ to obtain $Y_{1}$. In addition, a simple convolution operation: $Y_{2}={ℱ}_{1}(X_{2})=X_{2}*K_{1}$ is performed with the aim of preserving the original spatial context.

In the third step, the self-calibration scale space is processed (Self-Calibration).


$$\begin{array}{c} T_{1}=AvgPool_{r}\left(X_{1}\right) \end{array}$$ (10)

$$\begin{array}{c} X_{1}^{\prime }=Up\left({ℱ}_{2}\left(T_{1}\right)\right)=Up\left(T_{1}*K_{2}\right) \end{array}$$ (11)

$$\begin{array}{c} Y_{1}^{\prime }={ℱ}_{3}\left(X_{1}\right)\cdot \sigma \left(X_{1}+X_{1}^{\prime }\right) \end{array}$$ (12)

$$\begin{array}{c} Y_{1}={ℱ}_{4}\left(Y_{1}^{\prime }\right)=Y_{1}^{\prime }*K_{4} \end{array}$$ (13)

The feature $X_{1}\backslash )$ is down-sampled r times using average pooling (Eq. (10), then feature extraction and up-sampling (bilinear interpolation, Eq. (11), and the output feature $Y_{1}\backslash )$ is obtained by calibrating the features extracted by $K_{3}\backslash )$ convolution after the Sigmoid activation function. σ is the Sigmoid function, and Up\) is the up-sampling.

In the fourth step, the original scale feature space is processed, and the feature $X_{2}\backslash )$ is extracted by $K_{1}\backslash )$ convolution to get the feature $Y_{2}$; in the fifth step, the splicing operation is performed on the two scale space output features $Y_{1}\backslash )$, $Y_{2}\backslash )$ to get the final output feature Y\).

**Fig 9:**
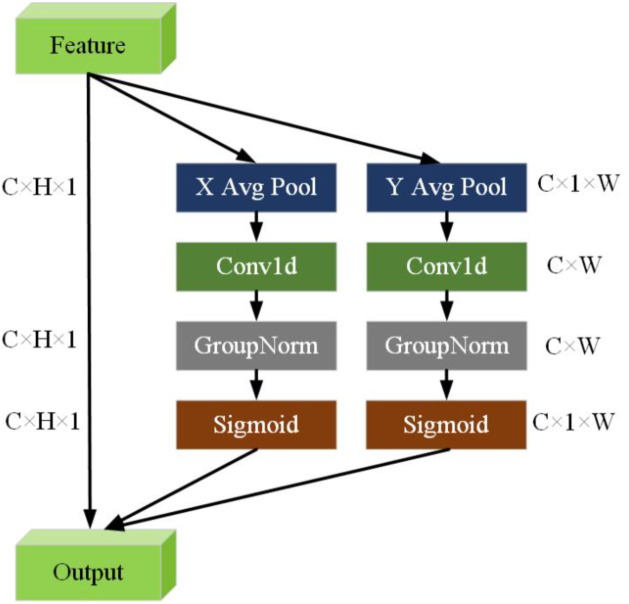
Structure of efficient localized attention mechanism.

In the feature extraction and classification process, there is no attention mechanism to differentiate feature importance, and equal weighting is applied across all feature types. When detecting surface defects on oversized rings, the detection model demonstrates a relatively weak ability to extract and characterize the features of small defects. Consequently, this paper introduces an efficient local attention (ELA) [34] mechanism between the neck and the head to acquire the input features of the CSP convolutional layer along the channel and spatial dimensions. The structure diagram of ELA is shown as Fig 9. The ELA module is similar to dilated convolution, which can skip some locations during attention calculation, thereby expanding the receptive field of each location. There is no need to store the entire attention weight matrix, which reduces memory requirements and enables coverage of a larger range at a smaller computational cost, greatly enhancing the network’s global capture capability. ELA employs fewer parameters and computations, performs adaptive feature fusion, and optimizes the extracted feature maps to identify valuable target regions, thereby enhancing the model’s focus on small targets amidst the dense information of target scene images. Parallel and serial fusion operations are applied to feature maps of different resolutions, increasing the perceptual field of the deep network’s extracted feature layer and enhancing the model’s feature extraction capability. This method achieves significant performance improvements with a simple structure. Similar to CA, ELA employs strip pooling to acquire feature vectors in the horizontal and vertical directions within the spatial dimension, maintains a narrow kernel shape to capture long-range dependencies, and prevents irrelevant regions from influencing label prediction, thus generating enriched features of the target location in respective directions.ELA processes these feature vectors independently in each direction to obtain the attention prediction, which is then combined using a dot product operation to ensure accurate location information for the region of interest.

The inherent limitations of conventional neck network architectures manifest in the attenuation or dissipation of fine-grained details during feature propagation and fusion. These minute details, however, are paramount for the accurate detection of diminutive objects. Furthermore, the constrained receptive fields of lower-level feature maps fail to encompass the entirety of crucial regions within these small targets. Original neck networks also lack dedicated optimization strategies tailored to the characteristics of small objects, relying on rudimentary feature fusion techniques, such as addition or concatenation, which prove inadequate to fully harness the complementary nature of multi-scale features, especially concerning the detection of small objects. Accordingly, this study introduces a novel multi-scale neck network architecture. By integrating feature information across diverse scales, it demonstrably augments the performance of the LMHD model in the detection of diminutive defects and those of varying dimensionalities. The incorporation of attention mechanisms into this modified neck network further enhances the process by assigning differential weights to feature maps across scales. This allows the model to adaptively discern and prioritize the most salient features, thereby achieving more efficacious feature fusion. Concurrently, the attention mechanism directs model focus toward regions containing small objects, amplifies their feature representation, and consequently elevates their detection rate. Ultimately, this proposed refinement empowers the model to better apprehend and capture objects of varying sizes and types, ultimately bolstering both the accuracy and robustness of the overall detection process.

### 3.6 Improvement of the loss function

In object detection, object localization relies on the bounding box regression loss. The Intersection over Union (IoU) loss function aims to align the predicted box with the actual object, enhancing the accuracy of target box localization. The YOLOv9 model’s loss function comprises both regression and classification losses. The classification loss is calculated using Binary Cross-Entropy Loss (BCE Loss), while the bounding box regression loss employs Complete IoU (CIOU). To improve network detection accuracy without increasing complexity, we focus on the bounding box regression loss function. The calculation formula of CIOU is as follows:


$$\begin{array}{c} CIOU=IOU-\frac{\rho^{2}}{c^{2}}-\alpha \vartheta \end{array}$$ (14)

$$\vartheta =\frac{4}{\pi^{2}}{\left(arcta\;n\;\frac{w_{gt}}{h_{gt}}-arcta\;n\;\frac{w_{pre}}{h_{pre}}\right)}^{2}$$ (15)

$$\begin{array}{c} \alpha =\frac{\vartheta }{1-IOU+\vartheta } \end{array}$$ (16)

The IOU in the above formula is the Intersection over Union loss, ρ is the distance of the center point of the rectangular box, c is the diagonal of the circumscribed rectangular box, and ϑ is the similarity of the aspect ratio, among which α is the influence factor of ϑ.

**Fig 10:**
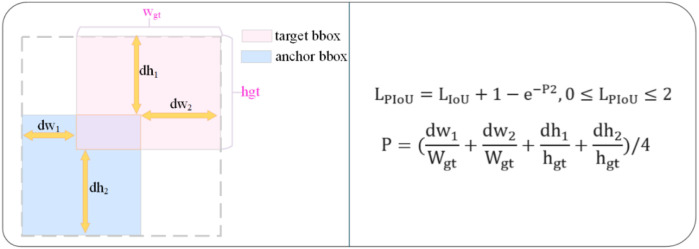
The PIoU loss of the bounding box regression.

The CIOU loss usually only considers the overlap area, center distance, and aspect ratio between the real box and the predicted box of the target. However, if only the difference of the aspect ratio is reflected in the loss instead of the actual relationship between w and $w_{gt}$ and h and $h_{gt}$, it will lead to a slowdown in the convergence rate of CIOU. And when the aspect ratios of the real box and the predicted box are very close, the penalty term ϑ of the aspect ratio in CIOU does not work, which will also affect the detection effect of the model. It is often difficult to adapt to the detection task of steel defect images in complex scenarios. To solve this problem, this paper adopts the PIoU v2 (Powerful-IoU v2) loss function, and Fig 10 shows the schematic diagram of the bounding box regression of the PIoU loss as shown in Fig 10 This function combines the target size adaptive penalty factor and the gradient adjustment function based on the quality of the anchor box. Thereby converging faster than the existing IoU-based losses.

The PIoU v2 loss is derived by adding an attention layer to the original PIoU loss, which enhances the focus on medium and high-quality anchor boxes, thereby improving the performance of the object detector. The attention function and the PIoU v2 loss formula are as follows:


$$\begin{array}{c} q=e^{-P},q\in (0,1] \end{array}$$ (17)

$$\begin{array}{c} u(x)=3x\cdot e^{-x^{2}} \end{array}$$ (18)

$$\begin{array}{c} L_{PIoU_{v}2}=u(\lambda q)\cdot L_{PIoU}=3\cdot (\lambda q)\cdot e^{-(\lambda q{)}^{2}}\cdot L_{PIoU} \end{array}$$ (19)

## 4. Experimental results and analysis

### 4.1 Dataset and preprocessing

**Fig 11:**
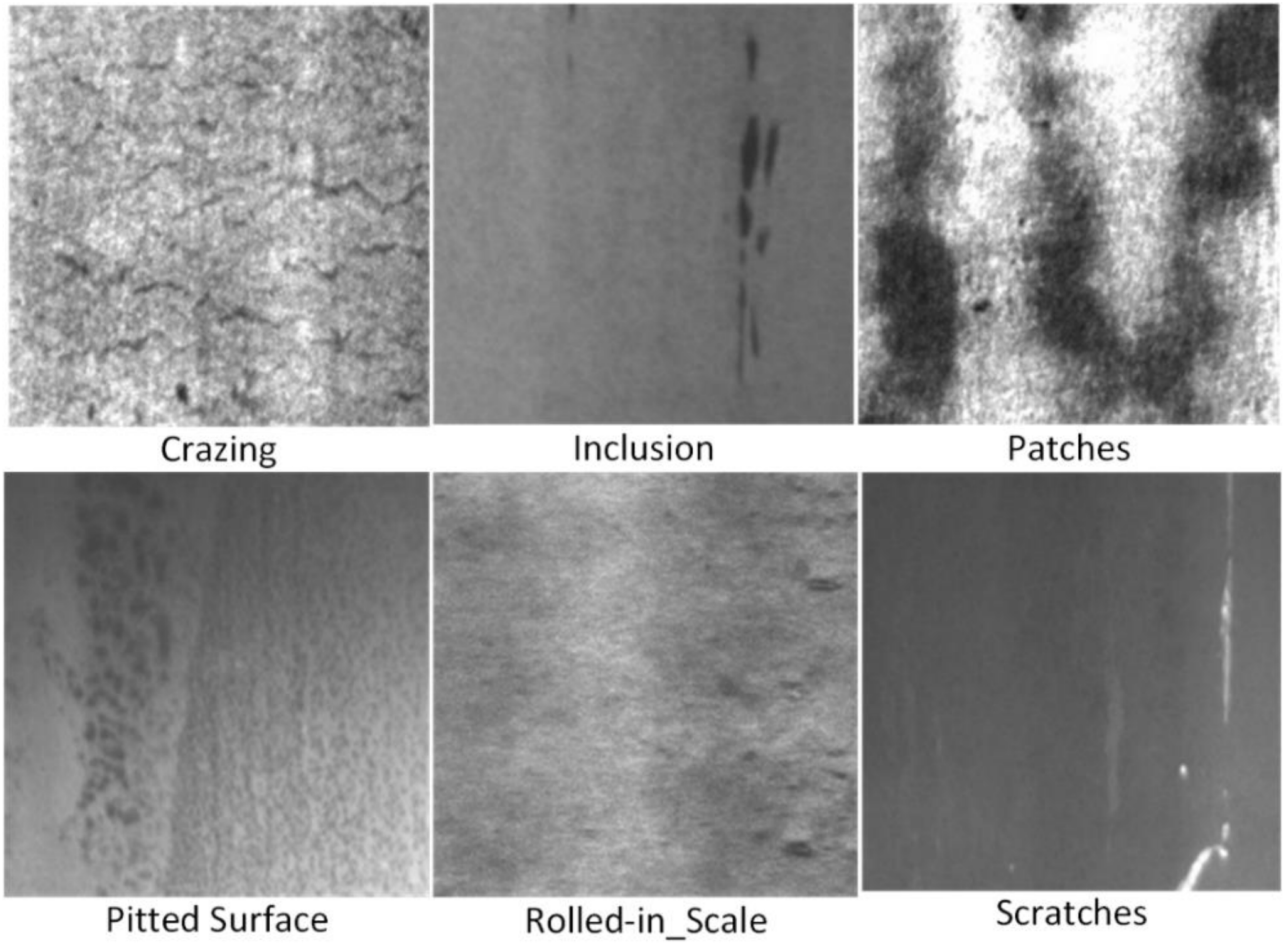
Steel surface defects.

The dataset used in this study is the NEU-DET [35] dataset from Northeastern University. To fully demonstrate the validity of our method, a small target remote sensing dataset was also selected for secondary verification. The dataset contains a total of 1,800 images in JPG format to validate the accuracy of the proposed model. The dataset includes six common types of surface defects: crazing (Cr), inclusions (In), patches (Pa), pitting surfaces (Ps), rolled-in scale (Rs), and scratches (Sc). The resolution of the dataset images is 200 x 200 pixels. Fig 11 provides example images of the six types of steel surface defects in the NEU-DET dataset. The dataset is randomly divided into training and test sets in a 9:1 ratio. It consists of 1,620 training images and 180 validation images.

### 4.2 Experimental parameter setting and model training

To conduct the experiments, we utilized the PyTorch deep learning framework to train the proposed model. The experimental environment included an NVIDIA GeForce RTX 4090D with 24 GB VRAM, Ubuntu 20.04 operating system, and Python 3.8. The experimental parameters are detailed in Table 1. We set the batch size to 10 and the image input size to 640 × 640 pixels. For the optimization algorithm, considering the gradient updating property during training, this study uses stochastic gradient descent (SGD). At the end of the model training, metrics such as model layers, number of parameters, giga floating point operations per second (GFLOPs), mAP@0.5, and mAP@0.5~0.95 are evaluated to assess the model’s performance.

**Table 1:** Hyperparameter settings.

Paremeter	Settings
**lr0 (initial learning rate)**	0.01
**lrf (final learning rate)**	0.01
**momentum**	0.937
**weight_decay**	0.005
**warmup_epochs**	3.0
**warmup_momentum**	0.8
**warmup_bias_lr**	0.1
**epochs**	220
**optimizer**	SGD
**mosaic**	1.0
**mixup**	0.15
**hsv_h(HSV-Hue augmentation)**	0.015
**hsv_s(HSV-Saturation augmentation)**	0.7
**hsv_v(HSV-Value augmentation)**	0.4

### 4.3 Evaluation of indicators

In a real production environment, the accuracy and speed of target detection are critical factors that affect productivity and quality. High miss rates and localization errors can degrade the quality of oversized rings, while slow detection speeds reduce production efficiency.

Therefore, in this experiment, we measure model performance using five evaluation criteria: recall (Recall), mean Average Precision (mAP), parameter count (Params), giga floating point operations per second (GFLOPs), and frames per second (FPS). Precision is the percentage of samples that are correctly identified as positive out of those predicted to be positive by the model. Recall is the ability of the model to identify all positive instances. Its formula is shown below:


$$\begin{array}{c} Precision=\frac{TP}{TP+FP} \end{array}$$ (20)

$$\begin{array}{c} Recall=\frac{TP}{TP+FN} \end{array}$$ (21)

Where FN denotes false negative case; TP denotes true case; and FP denotes false positive case. The curve drawn with the recall rate as the horizontal axis and the accuracy rate as the vertical axis is the PR curve, and the average value of the integrals under the curve is taken as the AP value for the category, whose formula is shown below:


$$\begin{array}{c} AP=\int_{0}^{1}\;p(r)dr \end{array}$$ (22)

The mAP is the sum of the mean accuracies of all tags divided by the total number of all categories, and its formula is shown below:


$$mAP=\frac{1}{C}\sum_{i=1}^{C}AP_{i}$$ (23)

### 4.4 Comparison experiments

To verify the balance between accuracy and speed of the proposed algorithm, Table 2 compares the model with current mainstream detectors, including the first-order detector SSD and the YOLO series (YOLOv5s, YOLOv5l, YOLOv7tiny, YOLOv8n, YOLOv8m, EfficientDet, YOLOv10l, YOLOv11l, YOLOv9c) as well as two-stage detectors (Faster R-CNN, Mask R-CNN), under consistent experimental conditions. By comparing performance with these mainstream models, the validity and superiority of the proposed model can be further verified. The NEU-DET dataset was employed to evaluate the results of this experiment. Our research model achieved an average accuracy of 87.0% (Precision and Recall values are shown in Fig 13), surpassing most detection algorithms and nearing the state-of-the-art level. LMHD outperforms YOLOv9c with a 7.8% increase in mAP, as shown in Table 2. While there is a slight difference in inference speed compared to YOLOv5s, LMHD achieves a 12.1% increase in detection accuracy and significantly improves inference speed compared to the baseline model. These improvements make LMHD highly suitable for industrial applications. Our proposed lightweight and efficient multi-scale cross-level component module reduces computational complexity, enhances model recall and accuracy, and lowers the leakage detection rate. Compared to the current SOTA models RT-DETR and YOLOv8, LMHD ensures lightweight performance while demonstrating significant advantages in detection accuracy. Compared to the two-stage representative algorithm Faster R-CNN, LMHD achieves a 9.0% increase in detection accuracy and an 84% reduction in the number of parameters. Compared to the one-stage representative algorithm SSD, LMHD improves detection accuracy by 14.6%. Among the comparison models, YOLOv5l achieves the highest detection accuracy of 81.9%. However, its large model size and slower inference speed limit its deployment and industrial application advantages. In contrast, the LMHD model offers excellent detection accuracy and faster inference time while being less than half the size of the YOLOv5l model.

**Table 2:** Test results comparison of different models.

Method	Recall/%	Params/M	mAP@0.5/%	GFLOPs/G	FPS
**Faster R-CNN**	92.3	108	78.0	307	27
**Mask R-CNN**	73.8	71.1	76.9	271.8	57
**RT-DETR**	73.2	19.9	72.4	57	56
**SSD**	66.8	24.7	72.4	62.4	29
**YOLOv5s**	74.7	7.0	74.9	15.8	220
**YOLOv5l**	73.4	46.1	81.9	197.7	33
**YOLOv7tiny**	66.4	6.0	74.0	13.1	165
**YOLOv8n**	73.1	3.0	78.9	8.2	208
**YOLOv8m**	68	25.8	80.4	78.7	72
**EfficientDet**	69	51.9	70.1	140	12
**YOLOv10l**	70.3	24.0	76.4	102.4	166.67
**YOLOv11l**	76.8	25.3	79.8	86.6	56.5
**YOLOv9c**	73.5	25.4	79.2	103.2	71.9
**LMHD(ours)**	76.8	17.9	87.0	58.9	70.4

**Fig 12:**
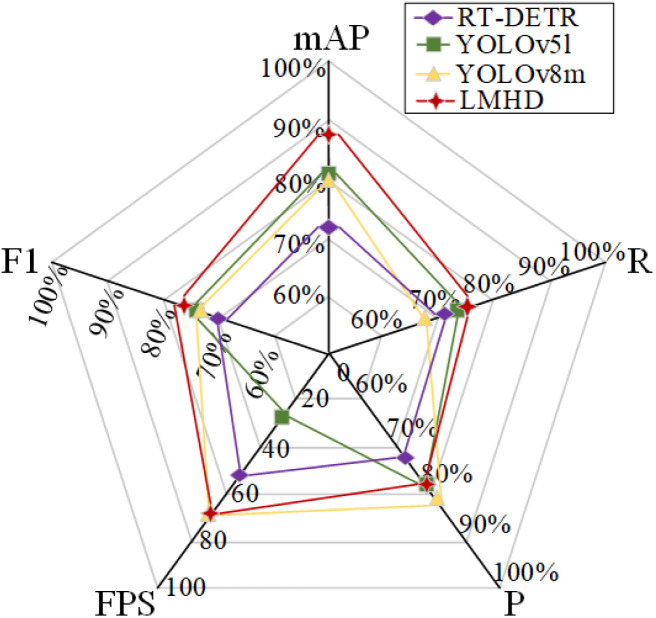
Comparison of the accuracy of each of the four detection models.

Regarding lightweight models for industrial deployment, only YOLOv5s, YOLOv7 tiny, and YOLOv8n exhibit smaller model sizes and parameters while maintaining good detection performance. And compared with the latest version of YOLOv10l, YOLOv11l, there are obvious advantages in model parameters and average detection accuracy, which further illustrates the practicality and effectiveness of the model in this study. As the second-most efficient detection model among the mainstream detectors, LMHD offers optimal detection performance while being lightweight. In conclusion, compared to other SOTA models, the LMHD model proposed in this paper has significant advantages and can be effectively deployed in real industrial environments.

To better understand the performance advantages of the proposed model, RT-DETR and the classical large model versions YOLOv5l and YOLOv8m are compared with the proposed LMHD model in terms of mAP (mean average precision), recall, accuracy, FPS (frames per second), and F1 (f-measure) metrics. The results are presented in Fig 12.

## 5. Ablation experiments

### 5.1 Improved modular ablation experiments

To comprehensively explore the detection performance and verify the effectiveness of each module in the proposed algorithm, a series of ablation experiments were conducted. The results of these experiments are presented in Table 3. The LMHD model algorithm proposed in this paper comprises three primary modules: RepViT, DVoVGSCSPM, and BiFPN. In our experiments, the neck network of the model was configured with BiFPN, including convolutional (conv), attention-guided convolutional (acconv), and Efficient Layer Aggregation (ELA) modules. The presence of a checkmark (√) under ‘conv’ signifies the utilization of a BiFPN architecture for the neck. A ‘√’ under ‘SCConv’ denotes that standard convolutions within the BiFPN were substituted with SCConv. Similarly, a ‘√’ under ‘ELA’ indicates the incorporation of an ELA module. Control experiments, in which the improved model utilized the original neck network structure, are identified by the absence of a ‘√’ under ‘BiFPN’. In this experimental setup, all three modules were evaluated for the LMHD detector. The presence of a module is indicated by a ‘√’ in the corresponding column. s shown in Table 3, replacing the network backbone with the lightweight RepViT network resulted in a reduction of the model’s parameters by 6.5M, accompanied by an increase in accuracy by 1.1%. However, this change led to a decline in the FPS by 38.9. Adding the DVoVGSCSPM module to the neck network resulted in a 0.2% decrease in detection accuracy. This reduction is attributed to the decreased model complexity and representational ability, which led to a decline in target detection ability. However, this change resulted in a 5% increase in FPS. Introducing the BiFPN network structure in the neck network decreased the number of parameters, while the FLOP increased. This is due to the BiFPN structure’s cross-level connections, which enhance the network’s ability to extract features at multiple scales. ntegrating all modules into the network decreased the model’s parameters by 7.5M, increased the mAP@0.5 by 6.9%, and increased the FPS by 24.3% compared to the previous state. The ablation experiments demonstrate that each enhanced module contributes to the network model’s performance enhancement to varying degrees. Combining the three improvement measures with the lightweight network model yields the lightweight and efficient LMHD algorithm. This algorithm improves the mAP@0.5 by 7.8% while significantly reducing the number of parameters by 30% compared to the original network model. In conclusion, the algorithm achieves an optimal balance between model complexity and performance in specific application scenarios. Fig 14 presents the comparison of the mAP@0.5 and loss function curves for each improvement module.

**Table 3:** Ablation experiments.

RepViT	DVOM		Bifpn			PIoU	Params/M	GFLOPs/G	mAP@0.5/%	FPS
	SimAM	DOConv	Conv	SCConv	ELA					
							25.4	103	79.2	71.9
√							18.9	65.5	80.3(↑1.1)	33
	√						16.9	45.9	79.8(↑0.6)	114.9
		√					21.8	80.2	84.7(↑5.5)	122.0
	√						21.8	80.2	81.6(↑2.4)	128.2
			√				24.3	127.5	78.3(↓0.9)	80
						√	25.4	103	83.7(↑4.5)	82.0
√	√						19.8	60.9	82.9(↑3.7)	94.3
√	√		√				11.1	51.2	82.7(↑3.5)	84.7
√			√				17.9	84.3	81.7(↑2.5)	30.5
	√		√				17.7	90.6	80(↑0.8)	102
√	√			√			17.9	58.9	85.6(↑6.4)	81.3
√	√		√		√		11.2	52.3	83.1(↑3.9)	140.8
√	√			√	√		18.5	64.1	81.5(↑2.3)	111.1
√		√		√	√		18.0	58.9	81.2(↑2.0)	108.7
√	√			√	√		17.9	58.9	86.1(↑6.9)	96.2
√	√			√	√	√	17.9	58.9	87.0(↑7.8)	70.4

### 5.2 Trunk ablation experiments

In order to fully verify the superiority of the lightweight backbone RepViT used in this paper, its performance is tested and compared with the current mainstream backbone networks, as shown in Table 4. Although the detection accuracy of the InceptionV4 backbone network is higher, this improvement is at the expense of the lightweight characteristics of the model, which is not applicable on the pursuit of lightweight improvement; the EfficientNetV2 backbone network has larger parameters than InceptionV4, but the detection accuracy is not enough, and the detection rate is not enough to satisfy the needs of practical industrial applications. MobileNetV4 is an efficient architecture designed for mobile devices, however, it only reduces 1.9 million parameters compared to the initial model, but the accuracy drops by 2.2%. SwinTransformer performs poorly on several parameters.Conformer backbone model is bulky and consumes a lot of computational resources, which is not suitable for lightweight network design. unsuitable for lightweight network design.The ShuffleNetV2 architecture significantly reduces the model size, with a 2% decrease in average detection accuracy and 8 million fewer model parameters compared to the initial backbone network of YOLOv9. However, the backbone network has a low FPS value, which is difficult to meet the real industrial detection requirements. SqueezeNet, developed by researchers at Berkeley and Stanford, is a lightweight detection model, but it underperforms on all parameters. The final experimental results show that RepVit reduces 6.5 million parameters, reduces computational cost by 34.5G, improves recall by 6.3%, and improves FPS by 27 compared to YOLOv9c. It demonstrates excellent performance and real-time performance, significantly improving detection accuracy while ensuring satisfactory detection speed. The algorithm achieves very satisfactory performance in the detection of surface defects on very large annular parts while significantly reducing complexity.

**Table 4:** Comparison of each lightweight backbone network.

Backbone network	Recall/%	mAP@0.5/%	Params/M	GFLOPs	FPS(GPU)
**YOLOv9c(baseline)**	72	79.2	25.4	103	71.9
**YOLOv9c-InceptionV4**	75.3	78.1(↓1.1)	32.8	83	82(↑10.1)
**YOLOv9c-EfficientNetV2**	73	71.2(↓8.0)	35.7	87.7	15(↓56.9)
**YOLOv9c-MobileNetV4**	75.1	77(↓2.2)	23.5	70.4	85(↑13.1)
**YOLOv9c-SwinTransformer**	70.1	72.4(↓6.8)	26.9	93.5	80(↑8.1)
**YOLOv9c-Conformer**	75.3	77.7(↓1.5)	105	300	30(↓41.9)
**YOLOv9c-ShuffleNetV2**	79.4	77.2(↓2.0)	17.4	60.5	19(↓52.9)
**YOLOv9c-SqueezeNet**	70	74(↓5.2)	22.4	64	20(↓51.9)
**Ours**	78.3	80.3(↑1.1)	18.9	65.5	46(↓15.9)

In this article, the abscissa is taken to be mAP@0.5, while the ordinate is FPS (GPU). Different color shapes represent the introduction of different types of backbone network models. The advantages of the backbone network used in this article are more intuitively presented in Fig 15.

### 5.3 Attention mechanism ablation experiments

To demonstrate the value of integrating the proposed model network structure with the enhanced ELA attention mechanism, this paper employs Gradient Weighted Class Activation Mapping (Grad-CAM) [36] to visualize and analyze the generated heat maps. The heat map provides a visual representation of the activation level of the model on the feature map, thereby offering an intuitive illustration of the model’s attention focus in different regions. As illustrated in Fig 16, Furthermore, it will combine CA (Coordinate attention) [37], CBAM (Convolutional Block Attention Module) [38], ECA (Efficient Channel Attention Network) [39], SimAM (Similaritv-based Attention Module), iRMB (lnverted Residual Mobile Block) [40], LSKblock (Large Selective Kernel Block) [41], and ELA (Efficient Local Attention mechanism). A comparison test is carried out by embedding seven different attention mechanisms in corresponding positions. The introduction of CoordAttention has the effect of improving the FPS of the model to a certain extent. However, the rest of the indicators are unsatisfactory. Furthermore, the introduction of the CBAM attention mechanism allows the model to pay attention to the channel and spatial features. However, the actual effect is not ideal. To enable the network to focus more on the channel information in the defective image, the ECA attention mechanism is introduced. This allows the network to automatically assign different weights to different channels when processing the feature map, thereby enabling the network to concentrate on the crucial features during the learning process and thus enhancing the model’s performance. However, the introduction of the ECA attention mechanism results in a decline in the model’s performance. In order to ensure that the model is lightweight, once more, we reintroduced SimAM into the network for experimental purposes.. The results demonstrated that the FPS of the model had been improved. In order to ascertain the efficacy of the model in carrying out intensive prediction of surface defects of oversized rings, we conducted experiments with the iRMB module. The results of these experiments were found to be unsatisfactory. The LSKblock module is capable of dynamically adjusting the spatial sensory field, and its introduction into the middle network model resulted in an improvement of the recall rate by 3.2%. However, the rest of the indicators remained unsatisfactory.

Ultimately, the incorporation of the ELA module into the proposed model demonstrates robust performance across all evaluated metrics, enhancing detection accuracy by 0.5% without requiring alterations to the underlying parameters. Table 5 illustrates the experimental data of the model following the incorporation of each attention mechanism. This presentation aims to clearly demonstrate the network model’s capacity to focus on the surface defects of the super-large ring components, both before and after enhancements.

**Table 5:** Comparison of improvements in attention mechanisms.

Algorithm model	Recall/%	mAP@0.5/%	Params/M	GFLOPs	FPS(GPU)
**Base model**	78.7	85.6	17.9	67	81.3
**+CoordAtt**	78	80.8(↓4.8)	18	73	104.2(↑22.9)
**+CBAM**	77	84.6(↓1.0)	18	70	90(↑8.7)
**+ECA**	71	79.4(↓6.2)	17.9	81.6	82.6(↑1.3)
**+ SimAM**	75.1	82.4(↓3.2)	17.9	69.6	112.4(↑31.3)
**+ iRMB**	76.6	83.1(↓2.5)	18.7	93.1	26.7(↓54.6)
**+ LSKblock**	81.9	82.8(↓2.8)	18.3	81.7	78.7(↓2.6)
**+ELA(ours)**	77.7	86.1(↑0.5)	17.9	73	96.2(↑14.9)

### 5.4 Improved loss function experiment

To investigate the relationship between the introduced PowerfulIoUv2 and model performance, we conducted comparative experiments by integrating EIoU, ShapeIoU, and CIoU from the initial model into the network. The results of the comparative experiments with different loss functions are shown in Table 6.

**Table 6:** Comparison of detection results with different loss functions.

Model	mAP/%	Params/M	FLOPs/G	FPS(GPU)
**+CIOU**	86.1	17.9	73	96.2
**+EIOU**	80.0	17.9	73	111
**+SHAPEIOU**	84.5	17.9	73	76.9
**+Powerfulliouv2**	87.0	17.9	73	70.4

The experimental results indicate that different loss functions have varying effects on the model, but they do not impact the model’s parameter count or computational load.

Compared to the original detection algorithm using the CIoU loss function, the LMHD algorithm with the improved loss function demonstrates a 0.9% increase in detection accuracy, reaching 87%, despite a reduction in detection speed.

Fig 17 demonstrates the comparison of loss functions for the LMHD model before and after improvement. The original model uses the CIoU loss function. Fig 17 shows that EIoU, which directly incorporates edge length as a penalty term, accelerates convergence speed. In contrast, ShapeIoU considers the shape and scale of edge regression samples, which further enhances the network’s convergence speed. PowerfulIoUv2 combines a target-size adaptive penalty factor with a gradient adjustment function that considers the quality of the anchor box, resulting in faster convergence compared to existing IoU-based losses. Additionally, it improves the mean Average Precision (mAP) of the network by 0.9%.

### 5.5 Visualization and analysis

To validate the detection performance of the LMHD model, we performed model inference experiments in this study. LMHD was compared with YOLOv5l, YOLOv8m, and RT-DETR. YOLOv5l, YOLOv8m, and RT-DETR are large-version deep-learning models that have shown good performance in previous studies, while RT-DETR is the current state-of-the-art (SOTA) model. In the inference experiments, six types of defects were randomly selected, and the prediction results of the three models were compared with the ground truth. As shown in Fig 18, LMHD showed the best performance compared to the other models in detecting steel surface defects. It achieved the highest detection accuracy in all six defect types and especially excels in detecting cracks and reducing missed detections. This advantage is mainly attributed to the DVoVGSCSPM module in LMHD, which enhances the model’s capability for multi-scale feature extraction and improves its detection of defects with complex shapes and smaller sizes, resulting in excellent defect detection performance under complex conditions.

**Fig 13:**
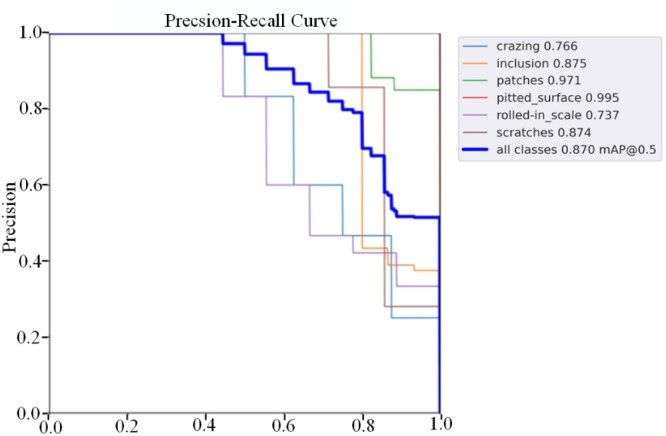
PR curve of NEU-DET on LMHD.

**Fig 14:**
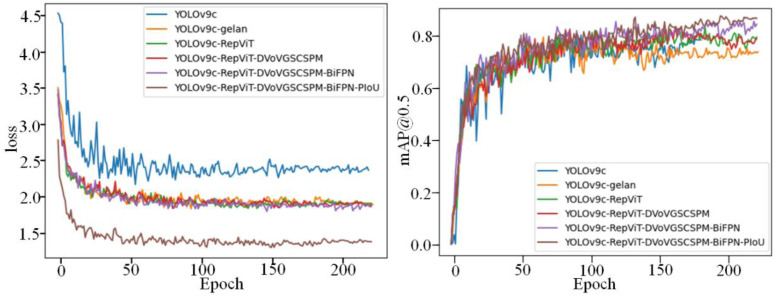
Comparison of mAP@0.5 and loss function curves of each improved module.

**Fig 15:**
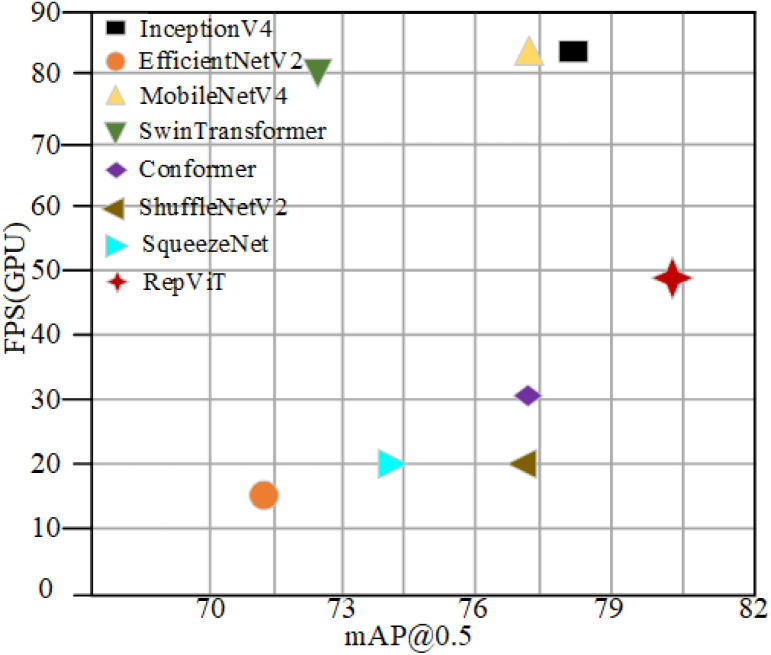
Comparison of the performance of each backbone network.

**Fig 16:**
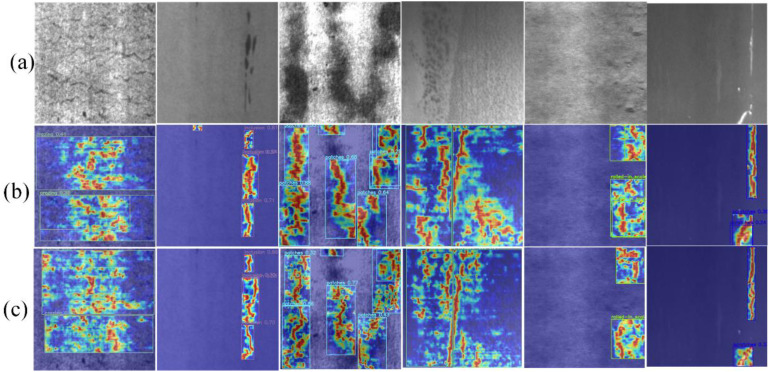
Grad-CAM visualization before and after improvement. **(a)** Initial image **(b)** Pre-improvement image **(c)** Post-improvement image.

**Fig 17:**
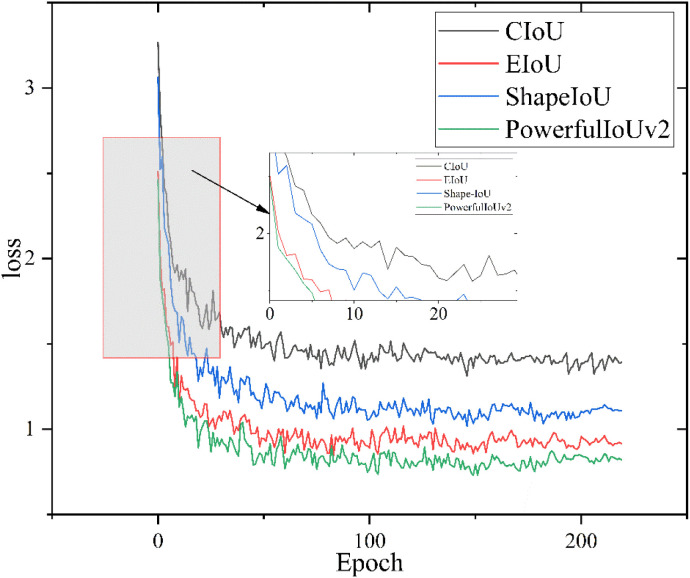
Comparison of loss functions.

**Fig 18:**
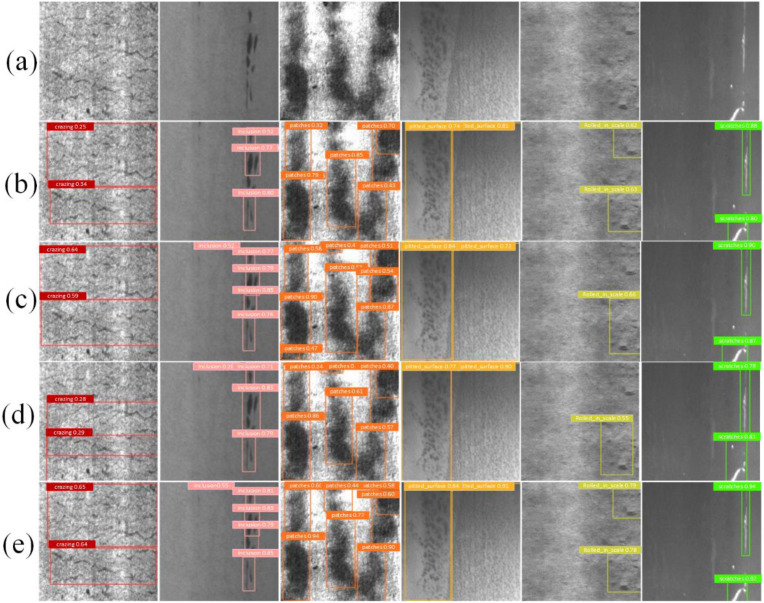
The prediction results of six types of defects. **(a)** Ground truth. **(b)** RT-DETR **(c)** YOLOv5l **(d)** YOLOv8m. **(e)** LMHD.

## 6. Conclusion

In order to improve the surface defect detection accuracy of the oversized rings, this paper proposes an end-to-end LMHD inspection network, and the following conclusions are drawn after experiments.

1. Through a large number of ablation experiments, the effectiveness of the improvement of each module is fully verified. That is, the algorithm in this paper adopts RepViT to act as the backbone of the model, adopts the improved BiFPN neck feature fusion network, in which the network uses the SCConv convolution and the ELA attention mechanism to fully extract the feature information, and employs the designed DVoVGSCSPM module, which enriches the semantic information of the feature map, effectively improves the feature extraction ability of the network, and greatly improves the CNN’s performance, and finally the use of PIoUv2 loss function is taken to accelerate the model convergence ability [42].
2. The LMHD detection algorithm is compared with various mainstream detection algorithms for experiments, and the LMHD obtains the highest detection accuracy, the best overall performance, and the detection accuracy of all types of defects is improved, which further proves the feasibility and practicality of the LMHD algorithm in the field of surface defect detection of oversized rings.
3. The experimental results show that the improvement of the LMHD algorithm proposed in this paper is effective, and the experimental comparison proves that the algorithm can further improve the accuracy of the detection of surface defects on the oversized rings, and also provides a new idea for the research in this field.

It is imperative to acknowledge that the proposed model continues to exhibit certain limitations. It is essential to consider the limitations of resources when implementing real- time detection on edge devices, necessitating the utilisation of lightweight and efficient algorithms. The algorithm presented in this paper is not without room for improvement in terms of model volume. The model demonstrated satisfactory performance, interpretability, and computational complexity; however, further enhancements are still warranted. Further research should concentrate on optimising the model and its practical application in industrial contexts.

## References

[1] Kim M, Dalhoff P eds. Yaw systems for wind turbines–overview of concepts, current challenges and design methods. In: Journal of physics: conference series. IOP Publishing; 2014.

[2] García-Martín J, Gómez-Gil J, Vázquez-Sánchez E. Non-destructive techniques based on eddy current testing. Sensors (Basel). 2011;11(3):2525–65. doi: 10.3390/s110302525 22163754 PMC3231639

[3] Amir YM, Thörnberg B. High precision laser scanning of metallic surfaces. Inter J Optics. 2017;2017:1–13. doi: 10.1155/2017/4134205

[4] Hull B, John V, Hull B, John V. Liquid penetrant inspection. In: Non-destructive testing. 1988. 7–17.

[5] Hao R, Lu B, Cheng Y, Li X, Huang B. A steel surface defect inspection approach towards smart industrial monitoring. J Intell Manuf. 2020;32(7):1833–43. doi: 10.1007/s10845-020-01670-2

[6] Luo Q, He Y. A cost-effective and automatic surface defect inspection system for hot-rolled flat steel. Robot Comp-Integ Manuf. 2016;38:16–30. doi: 10.1016/j.rcim.2015.09.008

[7] Wang H, Li Z, Wang H. Few-shot steel surface defect detection. IEEE Trans Instrum Meas. 2022;71:1–12. doi: 10.1109/tim.2021.3128208

[8] Zhang D, Hao X, Wang D, Qin C, Zhao B, Liang L, et al. An efficient lightweight convolutional neural network for industrial surface defect detection. Artif Intell Rev. 2023;56(9):10651–77. doi: 10.1007/s10462-023-10438-y

[9] Lecun Y, Bottou L, Bengio Y, Haffner P. Gradient-based learning applied to document recognition. Proc IEEE. 1998;86(11):2278–324. doi: 10.1109/5.726791

[10] Krizhevsky A, Sutskever I, Hinton GE. ImageNet classification with deep convolutional neural networks. Commun ACM. 2017;60(6):84–90. doi: 10.1145/3065386

[11] He K, Zhang X, Ren S, Sun J eds. Deep residual learning for image recognition. In: Proceedings of the IEEE Conference on Computer Vision and Pattern Recognition, 2016.

[12] Tan M, Le Q eds. Efficientnet: Rethinking model scaling for convolutional neural networks. In: International conference on machine learning. 2019.

[13] Zhu Y, Li G, Wang R, Tang S, Su H, Cao K. Intelligent fault diagnosis of hydraulic piston pump combining improved LeNet-5 and PSO hyperparameter optimization. Appl Acoustics. 2021;183:108336. doi: 10.1016/j.apacoust.2021.108336

[14] Marques G, Agarwal D, de la Torre Díez I. Automated medical diagnosis of COVID-19 through EfficientNet convolutional neural network. Appl Soft Comput. 2020;96:106691. doi: 10.1016/j.asoc.2020.106691 33519327 PMC7836808

[15] Michele A, Colin V, Santika DD. MobileNet convolutional neural networks and support vector machines for palmprint recognition. Procedia Computer Science. 2019;157:110–7. doi: 10.1016/j.procs.2019.08.147

[16] Ren S, He K, Girshick R, Sun J. Faster R-CNN: towards real-time object detection with region proposal networks. IEEE Trans Pattern Anal Mach Intell. 2017;39(6):1137–49. doi: 10.1109/TPAMI.2016.2577031 27295650

[17] Liu W, Anguelov D, Erhan D, Szegedy C, Reed S, Fu CY eds. Ssd: Single shot multibox detector. In: Computer Vision–ECCV 2016: 14th European Conference, Amsterdam, The Netherlands, October 11–14, 2016, Proceedings, Part I, 2016.

[18] Redmon J ed. You only look once: Unified, real-time object detection. In: Proceedings of the IEEE Conference on Computer Vision and Pattern Recognition, 2016.

[19] Ren Q, Geng J, Li J eds. Slighter faster R-CNN for real-time detection of steel strip surface defects. In: 2018 Chinese Automation Congress (CAC). IEEE; 2018.

[20] Shi X, Zhou S, Tai Y, Wang J, Wu S, Liu J eds. An improved faster R-CNN for steel surface defect detection. In: 2022 IEEE 24th International Workshop on Multimedia Signal Processing (MMSP). IEEE; 2022.

[21] Lu M, Sheng W, Zou Y, Chen Y, Chen Z. WSS-YOLO: An improved industrial defect detection network for steel surface defects. Measurement. 2024;236:115060. doi: 10.1016/j.measurement.2024.115060

[22] Gao S, Chu M, Zhang L. A detection network for small defects of steel surface based on YOLOv7. Digital Signal Processing. 2024;149:104484. doi: 10.1016/j.dsp.2024.104484

[23] Wang L, Liu X, Ma J, Su W, Li H. Real-time steel surface defect detection with improved multi-scale YOLO-v5. Processes. 2023;11(5):1357. doi: 10.3390/pr11051357

[24] Wang A, Chen H, Lin Z, Pu H, Ding G. Repvit: Revisiting mobile cnn from vit perspective. arXiv. 2023. https://arxiv.org/abs/2307.09283

[25] Categorization D, Koonce B. Convolutional neural networks with swift for tensorflow.

[26] Li H, Li J, Wei H, Liu Z, Zhan Z, Ren Q. Slim-neck by GSConv: a better design paradigm of detector architectures for autonomous vehicles. arXiv preprint. 2022. doi: 10.48550/arXiv.2206.02424

[27] Yang L, Zhang RY, Li L, Xie X eds. Simam: a simple, parameter-free attention module for convolutional neural networks. In: International conference on machine learning. PMLR; 2021.

[28] Cao J, Li Y, Sun M, Chen Y, Lischinski D, Cohen-Or D, et al. DO-Conv: depthwise over-parameterized convolutional layer. IEEE Trans Image Process. 2022;31:3726–36. doi: 10.1109/TIP.2022.3175432 35594231

[29] Lin TY, Dollár P, Girshick R, He K, Hariharan B, Belongie S eds. Feature pyramid networks for object detection. In: Proceedings of the IEEE conference on computer vision and pattern recognition. 2017.

[30] Wang K, Liew JH, Zou Y, Zhou D, Feng J eds. Panet: few-shot image semantic segmentation with prototype alignment. In: Proceedings of the IEEE/CVF International conference on computer vision. 2019.

[31] Tan M, Pang R, Le QV eds. Efficientdet: scalable and efficient object detection. In: Proceedings of the IEEE/CVF conference on computer vision and pattern recognition. 2020.

[32] Liu J, Mu J, Sun H, Dai C, Ji Z, Ganchev I. BFG&MSF-Net: boundary feature guidance and multi-scale fusion network for thyroid nodule segmentation. IEEE Access. 2024.

[33] Liu JJ, Hou Q, Cheng MM, Wang C, Feng J eds. Improving convolutional networks with self-calibrated convolutions. In: Proceedings of the IEEE/CVF Conference on computer vision and pattern recognition. 2020.

[34] Xu W, Wan Y. ELA: efficient local attention for deep convolutional neural networks. 2024. https://arxiv.org/abs/240301123

[35] He Y, Song K, Meng Q, Yan Y. An end-to-end steel surface defect detection approach via fusing multiple hierarchical features. IEEE Trans Instrum Meas. 2020;69(4):1493–504. doi: 10.1109/tim.2019.2915404

[36] Selvaraju RR, Cogswell M, Das A, Vedantam R, Parikh D, Batra D eds. Grad-cam: visual explanations from deep networks via gradient-based localization. In: Proceedings of the IEEE international conference on computer vision. 2017.

[37] Hou Q, Zhou D, Feng J eds. Coordinate attention for efficient mobile network design. In: Proceedings of the IEEE/CVF conference on computer vision and pattern recognition. 2021.

[38] Woo S, Park J, Lee J-Y, Kweon IS eds. Cbam: convolutional block attention module. In: Proceedings of the European conference on computer vision (ECCV). 2018.

[39] Wang Q, Wu B, Zhu P, Li P, Zuo W, Hu Q eds. ECA-Net: Efficient channel attention for deep convolutional neural networks. In: Proceedings of the IEEE/CVF conference on computer vision and pattern recognition. 2020.

[40] Zhang J, Li X, Li J, Liu L, Xue Z, Zhang B eds. Rethinking mobile block for efficient attention-based models. In: 2023 IEEE/CVF International Conference on Computer Vision (ICCV). IEEE Computer Society; 2023.

[41] Li Y, Hou Q, Zheng Z, Cheng MM, Yang J, Li X eds. Large selective kernel network for remote sensing object detection. In: Proceedings of the IEEE/CVF International conference on computer vision. 2023.

[42] Shi L, Zhou W, Wu Y, Yuan N, Zang X, Ji Z, et al. DCM-CNER: a dual-channel model for clinical named entity recognition based on embedded ConvNet and Gated Dilated CNN. IEEE Access. 2024;12:97726–38. doi: 10.1109/access.2024.3422677

